# Infants with microcephaly due to ZIKA virus exposure: nutritional status and food practices

**DOI:** 10.1186/s12937-019-0429-3

**Published:** 2019-01-11

**Authors:** Samira Fernandes Morais dos Santos, Fernanda Valente Mendes Soares, Andrea Dunshee de Abranches, Ana Carolina Carioca da Costa, Maria Elisabeth Lopes Moreira, Vania de Matos Fonseca

**Affiliations:** Institution: Instituto Nacional de Saúde da Mulher, Criança e Adolescente Fernandes Figueira – Fundação Oswaldo Cruz (IFF- FIOCRUZ), Av. Rui Barbosa, 716, Rio de Janeiro, 22250-020 Brazil

**Keywords:** Zika virus, Microcephaly, Nutritional status, Infant nutrition

## Abstract

**Background:**

Children with microcephaly due to vertical exposure to Zika virus are an interesting population for investigation. Highlighted among their unique aspects are those related to nutrition due to its impact on child growth and development. Knowledge about the nutrition of microcephalic infants can help mothers and caregivers provide better care. Thus, this study aimed to describe the nutritional status and feeding practices of infants with microcephaly due to Zika virus exposure at birth and 12–23 months of age.

**Methods:**

This is a descriptive study developed from a cohort of patients attending a public institution of reference. A total of 65 infants attended outpatient nutrition clinics. The food practices were described using the 24-h food recall and food consumption indicators. Anthropometric measurements and consultations were made using the Child Health Handbook to obtain information on the nutritional status (weight, height and head circumference) at the time of consultation and birth.

**Results:**

There was a significant decrease in z-scores for weight, height and head circumference (HC) from birth to the time of the consultation. However, most infants did not show weight-for-height deficits. Additionally, HC was correlated with the anthropometric indices weight-for-age, height-for-age, body mass index-for-age and weight-for-height.

**Conclusion:**

Infants exhibited a worsening of their nutritional status between birth and the time of their consultation, notably when we evaluated the indices of height and head circumference for age. The main inadequacies regarding dietary practices were low food diversity, use of ultra-processed products and low lipid intake.

## Introduction

Microcephaly is a condition in which the head circumference measurement is smaller than the typical range of values for children of the same age and gender [1, 2]. In 2015, there was an increased prevalence of microcephalic neonates in Brazil [3] related to the outbreak of the Zika virus infection during the gestational period, which established a new population [4] with a congenital infection to be investigated. Among the characteristics to be investigated in relation to these children, we highlight those related to nutrition due to its impact on children’s neurocognitive growth and development [5, 6], especially on children with neurological impairment [7].

Nutritional assessment is one of the components of child health care that can be encompassed by their primary care [8], and information related to the nutrition of infants with microcephaly can help mothers and health care providers.

Thus, this study aimed to describe the nutritional status and dietary practices of microcephalic infants vertically exposed to the Zika virus at birth and 12–23 months of age.

## Methods

This is a descriptive study developed with data from the cohort “*Vertical exposure to Zika virus and its consequences on the neurodevelopment of the child*”, carried out at the Fernandes Figueira National Institute of Women, Children and Adolescent Health – Oswaldo Cruz Foundation (IFF/FIOCRUZ), a reference institution for this population. The Research Ethics Committee of IFF/FIOCRUZ approved the project under Opinion CAAE 52675616.0.0000.5269.

During outpatient nutrition visits, infants aged 12–23 months with microcephaly due to vertical exposure to the Zika virus were included according to head circumference (HC) z-scores for age of less than two (− 2) standard deviations and/or based on medical findings in imaging studies of the central nervous system (transfontanelle ultrasonography and tomography). The maternal infection with Zika virus was considered in the presence of symptoms (skin rash, fever associated with arthralgia, myalgia, non-purulent conjunctivitis or headache) and positive result of the transcription polymerase chain reaction (RT- PCR) during pregnancy.

Based on interviews with the mothers/guardians, the following information was obtained:

1. Eating habits: a history of dietary practices; breastfeeding (BF) and exclusive breastfeeding (EBF) time; age of introduction of fruits, salty baby food (consistency - mashed/scraped or crushed/sieved), and foods such as sugar, soft drinks, processed juice and non-maternal milk.
2. Food consumption at the time of the consultation (12–23 months) from the food recall of 24 h. The foods/preparations reported by the mothers/guardians were entered into the DietPro® software for the calculation of total energy and macronutrient (protein, carbohydrate and lipid) values. The nutritional information contained in the labels of the processed foods was included when they were not present in the program. We also collected data on the frequency of meals, the consumption of farinaceous and dairy products, the route of administration of food (oral or gastrostomy) and the presence of choking and reflux evidenced by infants (reported by the mother/guardians).

This information on infant feeding was also used to construct five indicators proposed by Oliveira et al. [9] and the Ministry of Health [10]:

- Intake of at least one iron-rich food (egg, liver, lentils, beans, beef, chicken, pork, fish or other meat) [9];
- Consumption of at least one vitamin A-rich food (mango, papaya, pitanga, liver, carrot, broccoli, pumpkin or cabbage) [9];
- Consumption of some ultra-processed products (sugar, chocolate milk, curd cheese, margarine, coffee, canned foods, fried foods, soft drinks, natural guaraná, mate, candies, biscuits, processed juices, salty snacks, ice creams and popsicles, pies, jellies, cakes and other sweets) [9];
- Minimum dietary diversity: large meals (lunch and dinner) including one food from each group (cereals or tubers, vegetables, meats or eggs) and small meals including fruits and milk (including maternal milk) [9]; and
- Continuous breastfeeding (consumption of breast milk on the day before the interview) [10].

To calculate energy requirements, the formula proposed by Culley in 1969 was recommended for children with mental retardation and motor dysfunction [11]. Energy consumption corresponding to 90–110% of the recommended intake was adequate, and values below and above this range were deemed to be inadequate or excessive, respectively. The macronutrients were evaluated through the AMDR (acceptable macronutrient distribution range) [12], with the following percentages of the total energy value considered to be adequate: 5–20% of total dietary calories from proteins, 45–65% from carbohydrates and 30–40% from lipids. Intake values below and above these parameters were classified as deficient and excessive, respectively.

Anthropometry to evaluate nutritional status at 12–23 months (weight, height and head circumference) was measured according to the recommendations of the Food and Nutrition Surveillance System [13].

The anthropometric indicators weight-for-age (W/A), height-for-age (H/A), body mass index-for-age (BMI/A) and weight-for-height (W/H) were described according to the following stratification: z-scores < − 2 (lower than expected); z-scores ≥ − 2 and ≤ 2 (adequate for age); and z-scores > 2 (higher than expected). For the nutritional diagnosis at 12–23 months, W/H z-scores < − 2 were classified as a growth restriction. HC/A z-scores < − 3 were considered to indicate severe microcephaly [14] according to the WHO Growth Curves, 2006 [15]. The Anthro® version 3.2.2 program was used to calculate z-score values [16].

Birth information (date, gender, delivery type, gestational age, weight, height and HC) was collected by consulting the medical records and the Child Health Handbook, and the classification of the anthropometric indicators was performed as described in the previous paragraph, taking as reference the INTERGROWTH-21st curves [17].

Data were handled using descriptive statistics. Paired Student’s t-tests were used to verify the associations among the W/A, H/A and HC/A indices at birth and 12–23 months. We also tested the correlation between the current anthropometric index and Pearson’s correlation coefficient.

Data were typed and stored in a spreadsheet in Excel® version 2016 and analysed in SPSS 21, with a significance level of less than or equal to 0.05.

## Results

Sixty-five infants were included in the study. The infants had a mean age of 15 months, 50.8% were female, 54.8% were normal births, and 87.7% had a gestational age ≥ 37 weeks at birth.

We observed that most participants had adequate H/A and W/A z-scores at birth. Concerning nutritional status at 12–23 months, the anthropometric indices of W/A, BMI/A and W/H were within the parameters of adequacy for most infants. However, 56.9% had H/A deficits (Table 1). W/A, H/A and HC/A z-scores at birth and 12–23 months showed significant associations. There was a significant decrease in all mean z-scores between the periods of assessment (Table 2).

**Table 1:** Nutritional status of infants with microcephaly at birth and 12–23 months attending the IFF/FIOCRUZ, Rio de Janeiro, Brazil, June–December, 2017

Variable	Category	*n*	%
At birth			
Weight-for-age z-score	< −2	13	20
	≥ −2 and ≤ 2	52	80
Height-for-age z-score	< −2	15	23.1
	≥ −2 and ≤ 2	47	72.3
	> 2	3	4.6
Head circumference-for-age z-score	< −3	40	61.5
	≥ −3	25	38.5
At the time of the nutritional consultation (12–23 months)			
Weight-for-age z-score	< −2	27	41.5
	≥ −2 and ≤ 2	36	55.4
	> 2	1	1.5
Height-for-age z-score	< −2	37	56.9
	≥ −2 and ≤ 2	26	40
	> 2	1	1.5
Body mass index-for-age z-score	< −2	16	24.6
	≥ −2 and ≤ 2	44	67.7
	> 2	4	6.2
Weight-for-height z-score	< −2	19	29.2
	≥ −2 and ≤ 2	42	64.6
	> 2	3	4.6
Head circumference-for-age z-score	< −3	57	87.7
	≥ −3	8	12.3

**Table 2:** Differences between the means of weight-for-age, height-for-age and head circumference-for-age z-scores at birth and 12–23 months of infants with microcephaly treated at the IFF/FIOCRUZ, Rio de Janeiro, Brazil, June–December 2017

Variable	N	Mean	*P*-value^1^
W/A z-score^2^ at birth	64	−1.11	0.017
W/A z-score^2^ at the nutritional consultation^5^	64	−1.62	
H/A z-score^3^ at birth	64	−0.93	< 0.000
H/A z-score^3^ at the nutritional consultation	64	−1.94	
HC/A z-score^4^ at birth	65	−3.14	< 0.000
HC/A z-score^4^ at the nutritional consultation^5^	65	−5.43	

^1^Paired Student’s t-test; ^2^weight-for-age; ^3^height-for-age; ^4^head circumference-for-age; ^5^12–23 months of age

A significant correlation was identified between the values of HC/A z-scores and all anthropometric indices: H/A (correlation coefficient 0.606, *p* <  0.000), W/A (correlation coefficient 0.587, *p* <  0.000), W/H (correlation coefficient 0.377, *p* = 0.02) and BMI/A (correlation coefficient 0.314, *p* = 0.012).

Regarding the history of feeding practices, approximately 80% of the infants were not exclusively breastfed until the sixth month. Difficulties with breastfeeding were reported by 53.6% of the mothers. Among them, the mammary gland (33%) and hypogalactia (12.5%) were the most frequently mentioned. We found that these first foods were predominantly offered in “mashed and scraped” consistencies, as well as the first salty baby foods. Most participants had already tried sugar (Table 3).

**Table 3:** Prevalence of growth restriction (weight-for-height z-score < − 2 standard deviations) according to the history of feeding practices of infants with microcephaly attending IFF/FIOCRUZ, Rio de Janeiro, Brazil, June–December 2017

			Growth Restriction	
Variable and Categories	*n*	%	*N* (%)	
			Yes	No
Exclusive breastfeeding time				
< 6 months	50	78.1	15 (30)	35 (70)
≥ 6 months	14	21.9	4 (30.8)	9 (69.2)
Breastfeeding time				
< 12 months	47	74.6	14 (30.4)	32 (69.6)
≥ 12 months	16	25.4	5 (31.3)	11 (68.8)
Age at introduction of fruit				
< 6 months	29	46.8	12 (41.4)	17 (56.6)
≥ 6 months	33	53.2	6 (18.8)	26 (81.2)
Age at introduction of 1st salty baby food				
< 6 months	24	38.7	8 (33.3)	16 (66.7)
≥ 6 months	38	61.3	10 (73)	27 (27)
Age at introduction of 2nd salty baby food				
< 7 months	19	31.7	8 (42.1)	11 (57.9)
≥ 7 months	38	63.3	9 (23.7)	29 (76.3)
Salty baby food not yet introduced in feeding	3	5	1 (33.3)	2 (66.7)
Consistency of first fruits				
Mashed and/or scraped	42	70	14 (34.1)	27 (65.9)
Crushed and/or sieved	18	30	3 (16.7)	15 (83.3)
Consistency of first salty baby food				
Mashed	29	48.3	7 (25)	21 (75)
Crushed and/or sieved	31	51.7	9 (29)	22 (71)
Introduction of sugar before 2 years				
No	17	27	1 (5.9)	16 (94.1)
Yes	46	73	17 (37.8)	28 (62.2)
Introduction of soft drinks and/or processed juice				
No	46	74.2	13 (28.9)	32 (71.1)
Yes	16	25.8	5 (31.3)	11 (68.8)
Age at introduction of non-maternal milk				
< 12 months	59	95.2	16 (27.6)	42 (72.4)
≥ 12 months	3	4.8	2 (66.7)	1 (33.3)

At 12–23 months of age, few participants met the minimum dietary diversity criteria or were continuously breastfeeding. Ultra-processed foods were used by 52.3% of the participants. We found that many of the infants consumed fruit in less than two meals a day, and dairy foods were consumed more frequently. Most infants consumed farinaceous food twice or more a day and had adequate energy consumption. Regarding the distribution of macronutrients, we found that although participants’ overall protein and carbohydrate intake met the dietary recommendations, the lipid intake was poor in almost half of the participants (Table 4).

**Table 4:** Prevalence of growth restriction (weight-for-height z-score < −2 standard deviations) according to feeding practices of infants with microcephaly aged 12–23 months attending IFF/FIOCRUZ, Rio de Janeiro, Brazil, June–December 2017

			Growth Restriction	
Variables and Categories	*n*	(%)	*N* (%)	
			yes	no
Consumption of iron-rich foods^a^				
No	8	12.3	4 (50)	4 (50)
Yes	57	87.7	41 (73.2)	15 (26.8)
Consumption of vitamin A-rich foods^b^				
No	23	37	7 (30.4)	16 (69.6)
Yes	39	63	11 (28.2)	28 (71.8)
Minimum dietary diversity^c^				
No	47	72.3	16 (34)	31 (66)
Yes	18	27.7	3 (17.6)	14 (82.4)
Continuous breastfeeding				
No	53	81.5	16 (30.8)	36 (69.2)
Yes	12	18.5	3 (25)	9 (75)
Consumption of ultra-processed foods^d^				
No	31	47.7	7 (23.3)	23 (76.7)
Yes	34	52.3	12 (35.3)	22 (64.7)
Number of daily meals				
< 6	25	38.5	7 (28)	18 (72)
≥ 6	40	61.5	12 (30.8)	27 (69.2)
Number of meals with fruits				
< 2	45	69.2	15 (33.3)	30 (66.7)
≥ 2	20	30.8	4 (21.1)	15 (78.9)
Number of salty meals				
< 2	14	21.5	4 (28.6)	10 (71.4)
≥ 2	51	78.5	15 (30)	35 (70)
Number of dairy meals				
≤ 2	16	24.6	3 (18.8)	13 (81.2)
> 2	49	75.4	16 (33.3)	32 (66.7)
Number of farinaceous meals				
≥ 2	47	72	17 (37)	29 (63)
< 2	18	28	2 (11.1)	16 (88.9)
Food administration route				
Oral	60	92.3	18 (30.5)	41 (69.5)
Gastrostomy	5	7.7	1 (20)	4 (80)
Report of choking				
No	34	52.3	9 (26.5)	25 (73.5)
Yes	31	47.7	10 (33.3)	20 (66.7)
Report of reflux				
No	50	77	12 (24.5)	37 (75.5)
Yes	15	23	7 (46.7)	8 (53.3)
Adequate energy intake^e^				
Inadequate	19	32	8 (42.1)	11 (57.9)
Adequate or excessive	41	68	11 (26.8)	30 (73.2)
Adequate protein intake^f^				
Adequate	55	91.7	18 (32.7)	37 (67.3)
Excessive	5	8.3	1 (20)	4 (80)
Adequate lipid intake^f^				
Deficient	29	48.3	8 (27.6)	21 (72.4)
Adequate	27	45	10 (37)	17 (63)
Excessive	4	6.7	1 (25)	3 (75)
Adequate carbohydrate intake^f^				
Deficient	7	11.7	2 (28.6)	5 (71.4)
Adequate	46	76.6	16 (34.8)	30 (65.2)
Excessive	7	11.7	1 (14.3)	6 (85.7)

^a^Consumption of meat (beef, chicken, pork, fish or other)/liver/egg/beans/lentils

^b^Consumption of papaya/mango/pitanga/pequi/buriti/liver/pumpkin/carrot/broccoli/cabbage

^c^Consumption of two salty meals containing one food from each group (cereals or tubers, vegetables, meat or eggs, legumes) and consumption of fruits and milk (including maternal) in snacks

^d^Ingestion of sugar/chocolate milk/margarine/curd cheese/coffee/canned food/fried food/soft drinks/mate, natural guaraná/candies/cookies/salty snacks/processed juices/jellies/ice cream/popsicles, cakes/pies and other sweets

^e^Adequate energy intake: attendance of 90–110% of the estimated energy expenditure from Culley’s formula, 1969, deficient and excessive: below and above this percentage, respectively

^f^The following energy distribution of macronutrients against total energy intake was considered adequate: 5–20% of total calories from proteins; 45–65% from carbohydrates and 30–40% from lipids. Intake values below and above these percentages were classified as deficient and excessive, respectively

## Discussion

Our results indicate a deteriorating nutritional status of infants from birth to the time of the consultation (12–23 months). The most negatively affected anthropometric indices (*p* <  0.000) were H/A and HC/A, which showed significant declines, eventually changing the growth z-score channel. There was a correlation between the HC/A z-score and all other current anthropometric indices, mainly for H/A and W/A, showing that the weight and size of the skull and brain have a greater impact on these indicators. Del Campo et al. [18] also found similar correlations among HC/A, W/A and H/A scores at the birth of children with (probable or confirmed) congenital Zika virus syndrome, with and without microcephaly. In describing the growth of children with the same (presumed) syndrome from birth to the 8th month, Silva et al. [19] found monthly decreases in the z-scores for mean weight, height and especially HC, which continued to decline with time among cases both with and without microcephaly. However, in our findings, unlike the compromised anthropometric indicators above, at the moment of the consultation, W/H was proportional in more than 60% of infants; that is, most of these children did not evidence a deficit of this indicator. Thus, this population is characterized by a unique anthropometric profile such that their height and weight were affected. However, this population also showed harmony between these two parameters at the time of the consultation. Thus, the W/H index is an important parameter for the nutritional assessment of these infants.

Regarding dietary practices, approximately 78% of infants remained under EBF for less than six months. Before the first year of life, more than 90% of them were already consuming non-maternal milk. At the time of the nutritional consultation, less than 20% were continuously breastfeeding, whereas in children without microcephaly, this frequency at 12 months was approximately 35% [20]. The early weaning observed in our results can be explained, in part, by the difficulties with breastfeeding reported by mothers, especially vis-à-vis the mammary gland. This is to be expected, since children with microcephaly due to Zika virus exposure may show dysphagia from the third month [21] when changes in oral-motor coordination, swallowing and sucking make breastfeeding a challenging task [15].

In the introduction of complementary feeding, most infants consume the first salty baby food and fruits in “mashed and scraped” consistencies, adjusting to the consistencies recommended for children in this age group [22], despite the neurological impairment that implies adaptations in their dietary practice. An intake of fruits in less than two meals a day and a higher frequency of dairy consumption characterized a poorly diversified diet. A small percentage (27.7%) met the criteria of minimum dietary diversity [10]. A survey with data from more than 100 countries showed that only 29% of children between 6 and 23 months of age have minimal food variety [23]. This raises the challenge of how to change this situation, regardless of the health status of the child, paying particular attention to their dietary practices, considering the impact of adequate nutrition during the first thousand days of life on the child’s current and future health and life conditions [24].

In addition, we identified the introduction of the consumption of ultra-processed foods as inadequate from the point of view of nutritional quality, especially in children under two years of age, given their high levels of sodium, sugar and fat, high energy density and low nutritive content [25], which are associated with chronic noncommunicable diseases in adulthood [26]. The values found for the intake of these foods (52%) were close to those in the study by Marinho et al. [27] (58%) in children under two years of age without neurological impairment.

Most infants (68%) had an adequate or excessive energy intake. However, since the balance between the macronutrient percentage distribution, which is as important as the energy intake, is desired, we found that 50% of the babies had poor lipid intake. This is a factor of concern, given the importance of lipids and, more specifically, essential fatty acids, in child growth, as well as in visual and central nervous system development [28]. Considering that these systems may be affected by the congenital Zika virus syndrome [29, 30], the adequate consumption of lipids for these children exposed to the virus becomes even more crucial. The inadequate dietary intake of lipids may be explained by the main challenge to eating in this study (choking), when mothers thicken food through the use of carbohydrate-rich farinaceous food. These foods were used at least two times a day for more than 70% of the children, possibly displacing other lipid-rich foods, thus contributing to meeting the energy requirements.

A possible limitation of this study is the use of a single 24-h food recall, which does not consider dietary variability. However, this limitation may have been minimized by the food monotony of the age group and population studied. We hope that these results can provide insight into this stage of research, since there are still few studies in the literature that address the food consumption of these babies. We also hope that these findings help to provide initial subsidies for the elaboration of future care protocols, which can be used in the clinical practice of professionals involved in the healthcare of these children.

## Conclusion

In conclusion, the infants studied exhibited a deterioration in their nutritional status between the two assessment points, notably when we evaluated the H/A and HC/A indices. The main inadequacies related to dietary practices were low food diversity, ultra-processed consumption and low lipid intake. We then considered the efforts that must be invested to ensure that care for these children is provided by a team of interdisciplinary professionals. The nutritionist should have an essential role on this team, especially concerning the guidelines for an age-appropriate diet, which consider the limitations of this population and aim to minimize nutritional deficits and promote a better quality of life.
